# *QuickStats*: Age-Adjusted Rates* of Firearm-Related Suicide,^†^ by Race, Hispanic Origin, and Sex — National Vital Statistics System, United States, 2019

**DOI:** 10.15585/mmwr.mm7041a5

**Published:** 2021-10-15

**Figure:**
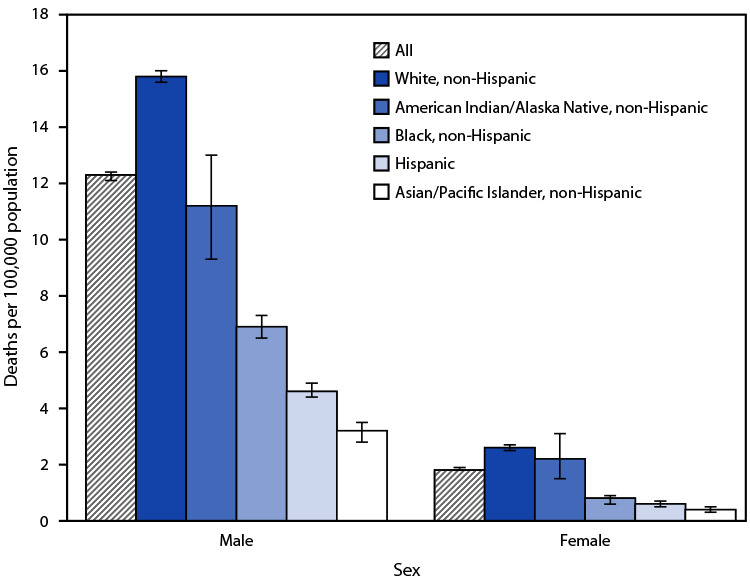
In 2019, among males, non-Hispanic White males had the highest age-adjusted rate of firearm-related suicide at 15.8 per 100,000 population, followed by non-Hispanic American Indian or Alaskan Native males (11.2), non-Hispanic Black males (6.9), Hispanic males (4.6), and non-Hispanic Asian or Pacific Islander males (3.2). Among females, non-Hispanic White and non-Hispanic American Indian or Alaskan Native females had the highest rates (2.6 and 2.2, respectively), followed by non-Hispanic Black females (0.8), Hispanic females (0.6), and non-Hispanic Asian or Pacific Islander females (0.4). Males had higher rates than females across all race and Hispanic origin groups.

**Source**: National Vital Statistics System, Mortality Data, 2019. https://www.cdc.gov/nchs/nvss/deaths.htm

For more information about this topic, CDC recommends the following link: https://www.cdc.gov/suicide/index.html.

